# A method for aligning RNA secondary structures and its application to RNA motif detection

**DOI:** 10.1186/1471-2105-6-89

**Published:** 2005-04-07

**Authors:** Jianghui Liu, Jason TL Wang, Jun Hu, Bin Tian

**Affiliations:** 1 Department of Biochemistry and Molecular Biology, New Jersey Medical School, University of Medicine and Dentistry of New Jersey, Newark, NJ 07101, USA; 2 Department of Computer Science, New Jersey Institute of Technology, University Heights, Newark, NJ 07102, USA

## Abstract

**Background:**

Alignment of RNA secondary structures is important in studying functional RNA motifs. In recent years, much progress has been made in RNA motif finding and structure alignment. However, existing tools either require a large number of prealigned structures or suffer from high time complexities. This makes it difficult for the tools to process RNAs whose prealigned structures are unavailable or process very large RNA structure databases.

**Results:**

We present here an efficient tool called RSmatch for aligning RNA secondary structures and for motif detection. Motivated by widely used algorithms for RNA folding, we decompose an RNA secondary structure into a set of atomic structure components that are further organized by a tree model to capture the structural particularities. RSmatch can find the optimal global or local alignment between two RNA secondary structures using two scoring matrices, one for single-stranded regions and the other for double-stranded regions. The time complexity of RSmatch is *O(mn) *where *m *is the size of the query structure and *n *that of the subject structure. When applied to searching a structure database, RSmatch can find similar RNA substructures, and is capable of conducting multiple structure alignment and iterative database search. Therefore it can be used to identify functional RNA motifs. The accuracy of RSmatch is tested by experiments using a number of known RNA structures, including simple stem-loops and complex structures containing junctions.

**Conclusion:**

With respect to computing efficiency and accuracy, RSmatch compares favorably with other tools for RNA structure alignment and motif detection. This tool shall be useful to researchers interested in comparing RNA structures obtained from wet lab experiments or RNA folding programs, particularly when the size of the structure dataset is large.

## Background

Ribonucleic acid (RNA) plays various roles in the cell. Many functions of RNA are attributable to their structural particularities (herein called RNA motifs). RNA motifs have been extensively studied for noncoding RNAs (ncRNAs), such as transfer RNA (tRNA), ribosomal RNA (rRNA), small nuclear RNA (snRNA), small nucleolar RNA (snoRNA), etc. [1]. More recently, small interfering RNA (siRNA) and microRNA (miRNA) have been under intensive studies [2]. Less well characterized are the structures in the un-translated regions (UTRs) of messenger RNAs (mRNAs) [3]. However, biochemical and genetic studies have demonstrated a myriad of functions associated with the UTRs in mRNA metabolism, including RNA translocation, translation, and RNA stability [4-6].

RNA structure determination via biochemical experiments is laborious and costly. Predictive approaches are valuable in providing guide information for wet lab experiments. RNA structure prediction is usually based on thermodynamics of RNA folding or phylogenetic conservation of base-paired regions. The former uses thermodynamic properties of various RNA local structures, such as base pair stacking, hairpin loop, and bulge, to derive thermodynamically favourable secondary structures. A dynamic programming algorithm is used to find optimal or suboptimal structures. The most well-known tools belonging to this group are MFOLD [7] and RNAFold in the Vienna RNA package [8,9]. Similar tools have been developed in recent years to predict higher order structures, such as pseudoknots [10]. On the other hand, RNA structure prediction using phylogenetic information infers RNA structures based on covariation of base-paired nucleotides [11-14]. It is generally believed that methods using phylogenetic information are more accurate. However, their performance critically depends on the high quality alignment of a large number of structurally related sequences.

Tools that align biosequences (DNA, RNA, protein), such as FASTA and BLAST, are valuable in identifying homologous regions, which can lead to the discovery of functional units, such as protein domains, DNA *cis *elements, etc. [15,16]. However, their success is more evident in the study of DNAs and proteins than of RNAs. This is mainly because the sequence similarity among DNAs and proteins can usually faithfully reflect their functional relationship, whereas additional structure information is needed to study the functional conservation among RNAs. Therefore, it is necessary to take into account both structural and sequential information in comparing RNA sequences.

Several tools are available that carry out RNA alignment and folding at the same time (Table 1). The pioneer work by Sankoff [17] involves simultaneous folding and aligning of two RNA sequences, and has huge time and space complexity (Table 1). FOLDALIGN [18] improves the Sankoff's method by (1) scoring the structure solely based on the number of base pairs, instead of the stacking energies; and (2) disallowing branch structures (junctions). Dynalign [19] reduces the time complexity by restricting the maximum distance allowed between aligned nucleotides in two structures. By taking into account local similarity, stem energy and covariations, Perriquet et al. [20] proposed CARNAC for pairwise folding of RNA sequences. Ji et al. [21] applied a graph-theoretical approach, called comRNA, to detect the common RNA secondary structure motifs from a group of functionally or evolutionally related RNA sequences. One noticeable advantage of comRNA is its capability to detect pseudoknot structures. In addition, algorithms using derivative-free optimization techniques, such as genetic algorithms and simulated annealing, have been proposed to increase the accuracy in structure-based RNA alignment [22-24]. For example, Notredame et al. [22] presented RAGA to conduct alignment of two homologous RNA sequences when the secondary structure of one of them was known. As shown in Table 1, most of these methods suffer from high time complexities, making the structure-based RNA alignment tools much less efficient than sequence-based alignment tools.

Tools that search for optimal alignment for given structures include RNAdistance [25], rna_align [26], and RNAforester [27]. RNAdistance uses a tree-based model to coarsely represent RNA secondary structures, and compares RNA structures based on edit distance. In a similar vein, rna_align [26] models RNA secondary structures by nested and/or crossing arcs that connect bonded nucleotides. With the crossing arcs, rna_align is able to align two RNA secondary structures, one of which could contain pseudoknots. RNAforester extends the tree model to forest model, which significantly improves both time and space complexities (Table 1). In addition, methods using Stochastic Context Free Grammars (SCFGs) have been developed to compare two RNA structures. Original SCFG models [28,29] require a prior multiple sequence alignment (with structure annotation) for the training purpose, thus their applicability is limited to RNA types for which structures of a large number of sequences are available, such as snoRNA and tRNA [28,30]. However, Rsearch [31] and stemloc [32], both based on SCFG, are capable of conducting pair-wise structure comparisons with no requirement for pre-alignment. Rsearch uses RIBOSUM substitution matrices derived from ribosomal RNAs to score the matches in single-stranded (ss) and double-stranded (ds) regions. stemloc uses "fold envelope" to improve efficiency by confining the search space involved in calculations. The time and space complexities of these two tools are also listed in Table 1. Furthermore, pattern-based techniques such as RNAmotif, RNAmot and PatSearch [3,33,34] have been used in database searches to detect similar RNA substructures. These tools represent RNA structures by a consensus pattern containing both sequence and structure information. One important advantage of these pattern-based tools is the ability of dealing with pseudoknots.

We present here a computationally efficient tool, called RSmatch, capable of both globally and locally aligning two RNA secondary structures. RSmatch does not require any prior knowledge of structures of interest. It can uncover structural similarities by means of direct aligning at the structure level. We demonstrate its application to database search and multiple alignment. We compared RSmatch with three widely used tools, PatSearch [35], stemloc [32] and Rsearch [31], demonstrating that RSmatch is faster or achieves comparable or higher accuracy than the existing tools when applied to a number of known RNA structures, including simple stem-loops and complex structures containing junctions.

## Implementation

### Secondary structure decomposition

**Figure 1:**
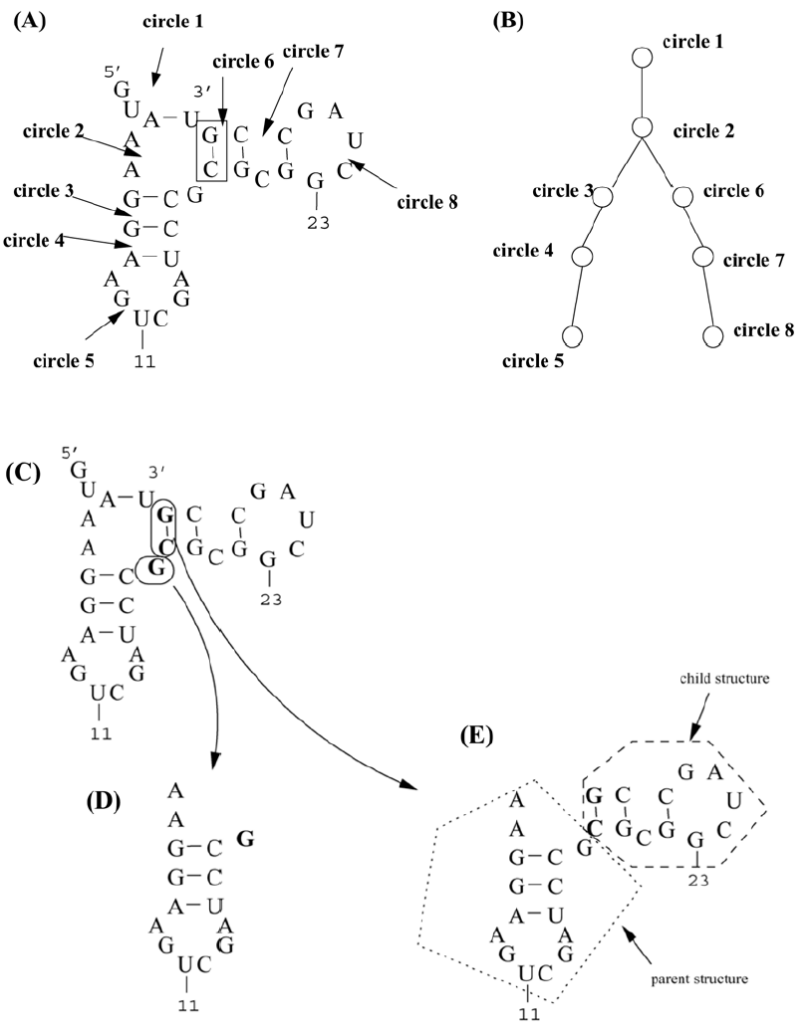
**RNA structure decomposition (A-B) and Partial structure determination (C-E). **(A) A hypothetical RNA secondary structure is decomposed into a set of circles. (B) The circles are organized into a hierarchical tree. As shown, circle 8 contains only one pair of bases that are bonded with each other; therefore it corresponds to a loop. Circle 7 contains two pairs of bases that are bonded with each other respectively and also contains a single base (nucleotide C); therefore circle 7 corresponds to a bulge. Circle 6 corresponds to a stem of length two since it does not contain any single base. Circle 2 contains more than two pairs of bonded bases; therefore it corresponds to a junction. (C) A hypothetical RNA secondary structure is used to illustrate how partial structures are determined. (D) The partial structure for the single base G in boldface is shown. (E) The partial structure for the base pair C-G in boldface consists of two parts, a parent structure and a child structure. The base pair itself is included in the child structure.

RSmatch models RNAs by a structure decomposition scheme similar to the loop model commonly used in the algorithms for RNA structure prediction [36,37]. With this model, pseudoknots are not allowed. Our method differs from the loop decomposition methods in that it completely decomposes an RNA secondary structure into units called circles (Figure 1A). When the secondary structure is depicted on a plane, a circle is defined as a set of nucleotides that are reachable from one another without crossing any base pair. As shown in Figure 1A, all circles are closed or ended by a base pair except the first circle (circle one in the Figure 1A), which always contains the 5'-most and the 3'-most bases. Various types of RNA structures, such as bulge, loop, and junction can be represented by circles, as shown in Figure 1A.

Circles of an RNA structure can be organized as a hierarchical tree according to their relative positions in the secondary structure, where each tree node corresponds to a circle (Figure 1B). This tree organization is informative to deduce the structural relationship among circles and reflects the structure particularities of the given RNA secondary structure. If two circles reside on the same lineage (path) in the tree, the circle appearing higher in the tree is called an ancestor of the other, and the latter is a descendent of the former. As a result, in the context of the hierarchical tree, two distinct circles fall into one of the following two categories, in the order of decreasing closeness: (i) the two circles maintain an ancestor/descendent relationship, or (ii) they share a common ancestor in the tree. For example, in Figure 1B, circle 2 is an ancestor of circle 5, whereas circle 6 does not have ancestor/descendent relationship with circle 5 since they are not on the same lineage. The double-stranded region or stem of a structure is decomposed into a set of "degenerated" circles, each containing only two base pairs. As such, a stem of *n *bases in length will result in *n *- 1 consecutive degenerated circles. Since a base pair may have two associated circles; we name one circle "the parent circle" and the other "the child circle" according to their positions in the hierarchical tree. For example, for the boxed C-G base pair in Figure 1A, circle 2 is its parent circle and circle 6 is its child circle.

### Structure alignment formalization

Given an RNA secondary structure, we consider two types of *structure components*, single bases and base pairs, in the secondary structure. To integrate both sequence and structure information, we introduce two constraints among the structure components: *precedence constraint *and *hierarchy constraint*. The precedence constraint is defined based on the precedence order among structure components and the hierarchy constraint specifies the inter-component relationship in the context of the hierarchical tree described above. The precedence order is determined by the 3' bases of individual structure components: the one with its 3' base closer to the RNA sequence's 5'-end precedes the other. For example, in Figure 1A, the single base component U (marked as the 11^th ^nucleotide) in circle 5 precedes the base pair component C-G (boxed) in circle 6.

To capture the inter-component relationship within the hierarchical tree context, we need to map each structure component to a circle in the tree. It is obvious that each single base can be mapped to a unique circle. However, a base pair could be mapped to two alternate circles: one parent circle and one child circle. To resolve this ambiguity, we always require mapping to the parent circle. The inter-component relationship is then reduced to the inter-circle relationship of three types: (i) ancestor/descendent, (ii) common ancestor, and (iii) identical circle.

Given two RNA secondary structures *A *and *B*, where *A*, referred to as the query structure, has *m *structure components {*A*^1^, *A*^2^, ..., *A*^*m*^} and *B*, referred to as the subject structure, has *n *structure components {*B*^1^, *B*^2^, ..., *B*^*n*^}, the structure alignment between *A *and *B *is formalized as a conditioned optimization problem based on the above two constraints: given a scoring scheme consisting of two matrices, one for matching two single bases and the other for matching two base pairs, find an optimal alignment between the two sets of structure components such that the aforementioned precedence and hierarchy constraints are preserved for any two matched component pairs (*A*^*i*^, *B*^*i*^) and (*A*^*j*^, *B*^*j*^). In other words, the two structure constraints between *A*^*i *^and *A*^*j *^must be respectively equivalent to that between *B*^*i *^and *B*^*j*^. This formalization has an implicit biological significance in that a single stranded region in one structure, if not aligned to a gap as a whole, will always align with a single stranded region in the other structure. This alignment requirement is important because single stranded regions are usually treated as functional units in binding to specific proteins.

### Algorithmic framework

A dynamic programming algorithm is employed in RSmatch. As with sequence alignment, the structure alignment could be either global or local. The difference lies only in the setup of initialization conditions; the algorithmic framework is the same since both global and local alignments must preserve the two constraints described above.

A scoring table is established with its rows/columns corresponding to the structure components of the two given RNA secondary structures. We organize the rows/columns in such a way that the precedence and hierarchy constraints are combined and easy to follow in the course of alignment computation. Specifically, we sort the structure components of each structure according to the precedence order defined above. It is straightforward that this arrangement of rows/columns makes the precedence constraint automatically preserved. However, preservation of the hierarchy constraint is much more complicated and can only be accomplished in the derivative analysis for each cell (entry) in the scoring table. We will discuss the derivation when filling in the scoring table.

Each cell of the scoring table represents an intermediate comparison between two *partial structures *corresponding to the cell's row and column components (either single base or base pair) respectively. The partial structure with respect to a structure component *c *(single base or base pair) is a set of structure components *S*_*c *_such that for any component *a *∈ *S*_*c*_, the following three structure constraints between *c *and *a *must be satisfied: (i) *a *precedes *c*; (ii) by the hierarchy constraint, *a *is not an ancestor of *c*; and (iii) *c *itself is included in *S*_*c*_.

Furthermore, since a base pair could appear in two circles, its corresponding partial structure could be divided into two smaller substructures: *parent structure *and *child structure*. Formally, given a base pair component *c*, the *parent structure *of *c *is the set of structure components *P*_*c *_⊆ *S*_*c *_(excluding *c *itself) such that for any component *a *∈ *P*_*c*_, *a*'s 3'-base is always 5' upstream of *c*'s 5'-base; the *child structure *of *c *is the set of structure components *L*_*c *_⊆ *S*_*c *_(including *c*) such that for any component *a *∈ *L*_*c*_, *a*'s 5'-base is always 3' downstream of *c*'s 5'-base. It can be shown that *P*_*c *_∪ *L*_*c *_= *S*_*c *_and *P*_*c *_∩ *L*_*c *_= *φ*. Examples of partial structures are given in Figure 1C–1E. As shown in Figure 1C, for a base pair, its child and parent structures together constitute the whole partial structure for the base pair.

As we will see in the following discussions, the concept of a partial structure and its byproducts (parent structure and child structure) form the kernel of our algorithmic framework. We can solve the RNA structure alignment problem progressively by aligning small structures and expanding each of them one structure component at a time until all structure components are covered.

### Preliminaries

Cells in the scoring table are processed row by row from top to bottom and from left to right within each row. By considering the row/column components, we have three types of cells: (i) a cell corresponding to two single bases; (ii) a cell corresponding to one single base and one base pair; and (iii) a cell corresponding to two base pairs. For (i), each cell stores the score of aligning the partial structures corresponding to the cell's row and column components respectively. For (ii) and (iii), we need to consider alignments involving the partial and child structures induced by the base pair components. Notice that the parent structures of the base pair components are excluded. It can be shown that each parent structure *P*_*c *_of component *c *can always be considered as the partial structure *S*_*x *_of some other component *x*, which means we only need to consider child and partial structures in the alignment computation. Consequently, the above three types of cells have one, two and four alignment scores respectively.

A scoring scheme is required to score the match of two structure components. We define the scoring scheme as a function *g*(*a*, *b*) where *a *and *b *represent two structure components that are matched with each other. Another important aspect of the alignment algorithm is to penalize the match involving gap(s). In the course of computation, one structure component (single base or base pair) could match with a gap or a whole small structure (parent or child structure) could match with a large gap. Intuitively, the larger the gap is, the heavier the penalty will be. In our implementation, we set an atomic penalty value, denoted as *u*, for the smallest gap equivalent to a single base. The penalty value for a large gap is proportional to its size in terms of the number of bases matched with the gap.

Let A* be a small structure in the query RNA structure A and B* a small structure in the subject RNA structure B. The score obtained by aligning the two structures A* and B*, denoted as *f*(A*, B*), is , where *G *represents the total number of gaps in aligning A* and B*.

### Initialization

We assume that the row components (*a*'s) are from the query RNA structure A and the column components (*b*'s) from the subject RNA structure B. We focus on global alignment here; initializations for local alignment can be derived similarly. The initialization conditions deal with the cases where at least one of the structures under alignment is an empty structure *φ*. This is equivalent to setting up the 0^th ^row/column in the scoring table. As discussed above, each base pair component has two small structures to be considered: a child structure and a partial structure. Thus, the aforementioned three types of cells have one, two and four initialization scores respectively.

For a given structure component *x *(single base or base pair), let *S*_*x *_represent its partial structure. If *x *is a base pair, we also use *L*_*x *_to represent its child structure. We have *f*(*φ*, *φ*) = 0. Furthermore, for any structure components *a *and *b*, *f*(*S*_*a*_, *φ*) = |*S*_*a*_|·*u*, *f*(*φ*, *S*_*b*_) = |*S*_*b*_|·*u*, if *a *and *b *are base pairs, *f*(*L*_*a*_, *φ*) = |*L*_*a*_|·*u *and *f*(*φ*, *L*_*b*_) = |*L*_*b*_|·*u *where |·| represents the cardinality of the respective set.

### Filling in the scoring table

The simplest cell type is the one whose row (column, respectively) component is a single base *a *(single base *b*, respectively). Let *a*^*p *^denote the structure component that precedes *a *by precedence order established before. Formally, in matching the partial structure *S*_*a *_with the partial structure *S*_*b *_there are only three possibilities: (i) *a *is aligned with *b*; (ii) *a *is aligned with a gap; and (iii) *b *is aligned with a gap. Thus the score of matching *S*_*a *_with *S*_*b *_can be calculated by Equation (1).



The second cell type is the one formed by one single base and one base pair. There are actually two symmetric subtypes where either *a *or *b *is a base pair. Since the analysis is identical, we only focus on the former case where *a *is a base pair. As discussed before, besides the partial structure *S*_*a *_we have to consider the child structure *L*_*a *_for the base pair *a*. Thus, for this type of cells, we have to compute two alignment scores.

**Figure 2:**
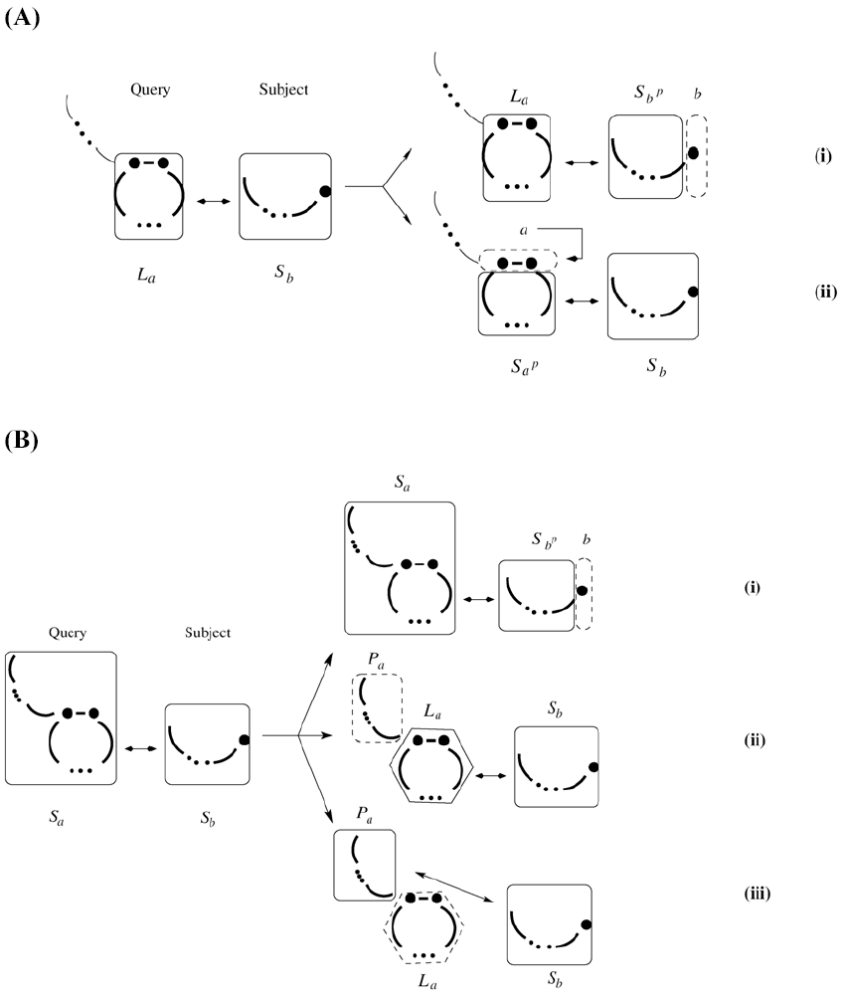
**Optimal structure alignment derivation. **(A) Structure alignment between the child structure *L*_*a *_in the query and the partial structure *S*_*b *_in the subject. The substructures enclosed by dashed lines are to be inserted/deleted and the substructures enclosed by solid lines are to be matched. (B) Structure alignment between the partial structure *S*_*a *_in the query and the partial structure *S*_*b *_in the subject. The substructures enclosed by dashed lines are to be inserted/deleted and the substructures enclosed by solid lines are to be matched.

By the principle of dynamic programming, the smaller size problem needs to be solved before the larger size problem. Thus we first find the structure alignment between the child structure *L*_*a *_and the partial structure *S*_*b*. _There are only two possibilities: (i) the single base component *b *is aligned with a gap; and (ii) the base pair *a *is aligned with a gap (see Figure 2A). Therefore we have



In aligning the partial structure *S*_*a *_with the partial structure *S*_*b*, _to preserve precedence and hierarchy constraints simultaneously, there are only three possibilities: (i) the single base *b *matches with a gap; (ii) the partial structure S_*b *_matches with the child structure *L*_*a*_; (iii) the partial structure S_*b *_matches with the parent structure *P*_*a *_(see Figure 2B). Thus,



For the third cell type, *a *is a base pair and *b *is a base pair. We need to compute four alignment scores because each base pair corresponds to two structures: one child structure and one partial structure. While aligning the child structure *L*_*a *_with the child structure *L*_*b*_, it is clear that



since both *a *and *b *are the last components in the respective child structures by precedence order. Equation (5) gives the alignment score between the partial structure *S*_*a *_and the child structure *L*_*b*_.



The first case corresponds to that *b *is aligned with a gap. If *b *does not match with a gap, it can be shown that, to preserve both precedence and hierarchy constraints, the second and third cases in Equation (5) cover all possible situations. Similarly, we can calculate the score of aligning the child structure *L*_*a *_and the partial structure *S*_*b *_as shown in Equation (6).



In aligning the partial structure *S*_*a *_with the partial structure *S*_*b*_, there are five possibilities: (i) the parent structure *P*_*a *_is matched with the parent structure *P*_*b *_and the child structure *L*_*a *_is matched with the child structure *L*_*b*_; (ii) the child structure *L*_*a *_is matched with gaps; (iii) the child structure *L*_*b *_is matched with gaps; (iv) the parent structure *P*_*a *_is matched with gaps; and (v) the parent structure *P*_*b *_is matched with gaps. Therefore



### Data sets

All experiments (unless otherwise specified) were carried out on a Linux system with two 2.4 GHz Intel processors and 3 GB memory. A human UTR structure database was constructed as follows. We downloaded 19,986 human RefSeq mRNA sequences (January 2004 version) from National Center for Biotechnology Information (NCBI). Each RefSeq sequence containing UTR regions, as indicated by RefSeq's GenBank annotation, was processed to extract its 5'UTR and 3'UTR sequences. For each UTR sequence, we took a 100 nt subsequence at every 50th nucleotide position from 5' to 3', making consecutive subsequences overlap with one another on a 50 nt segment. Subsequences shorter than 100 nt, e.g. at the 3' end, were also kept. Using the Vienna RNA package's RNAsubopt function with setting "-e 0", we then folded all obtained sequences to form the structure database. For any given RNA sequence, the setting "-e 0" resulted in multiple RNA structures all having the minimum free energy. The final database contained ~575,000 RNA secondary structures.

The structural patterns of a histone 3'UTR stem-loop structure (HSL3) and an iron responsive element (IRE) were used in this study, based on their specifications in the UTRdb database [3]. Three tools, PatSearch [38], stemloc [39] and Rsearch [40], were employed for comparison purposes. The efficiency of these tools was measured by CPU running time. The performance of each program was assessed by specificity and sensitivity. Specificity was calculated as TP/(TP + FP) and sensitivity as TP/(TP + FN), where TP was the number of true positives, FP the number of false positives, and FN the number of false negatives.

To test the applicability of RSmatch to complex structures, we downloaded RNA families from Rfam [1]. We only chose those families that had more than 10 seed RNAs and its consensus sequence length is no longer than 250 nucleotides. We had 64 families in the final data set. For each family, we randomly selected one member RNA as the query RNA and obtained its structure from Rfam. We then randomly chose 10 subject RNAs in the same family. Here we intentionally introduced noise by extending each subject RNA sequence with its adjacent sequences at both 3' and 5' ends to make the total length three times its original one.

## Results

### Studies of stem-loop structures in UTRs

Using our proposed algorithm, we first studied RNA motifs in UTR regions of human mRNA sequences. A well-known fact is that the accuracy and efficiency of RNA folding programs will decrease significantly when the sequences to be folded become very long. Satisfactory performance is usually obtained when the sequences have moderate lengths, i.e. one hundred nucleotides. Thus, we used a moving window scheme to get subsequences of 100 nt and folded them using the Vienna RNA package (see Implementation). In the RSmatch package, this subsequence length is a user-defined parameter.

Since the nucleotide conservation in the single-stranded region of an RNA sequence may differ from that in the double-stranded region, we used two scoring matrices, one for substitutions among single bases and the other among base pairs. This type of scoring scheme was also used in other studies [31,41]. Theoretically, the scoring matrix for single bases is a 4 × 4 table for all types of substitutions of single nucleotides, and the one for base pairs is a 16 × 16 table for all types of substitutions of base pairs. However since we used the Vienna RNA package, only six types of base pairs were observed in our studies, i.e. Watson-Crick base pairs A-U, U-A, G-C, C-G, and wobble base pairs G-U and U-G. Values used in the two matrices were empirically chosen so as to conform to the general understanding of the sequence and structure conservation of RNA motifs, as follows. (1) Mutations in the double-stranded region may not be detrimental to RNA's function if the mutated sequence still preserves the same secondary structure. Therefore base pair substitutions were rewarded with a positive score, instead of a penalty. (2) A sequence in the single-stranded region may be important for RNA's function, such as binding to proteins, and thus mismatches were penalized. To process gaps we used an arbitrary function *u *× *l*, where *u *was the atomic penalty value for a gap that is one single base long and *l *is the length of the gap in terms of the number of bases matched with the gap. In our experiments otherwise stated explicitly, the *u *was empirically set to -6 and changing the *u *value did not change the qualitative conclusion made in the paper provided that the absolute value of *u *was greater than any positive score in the scoring matrices. Users can freely change the *u *value when applying RSmatch to their own data set.

**Figure 3:**
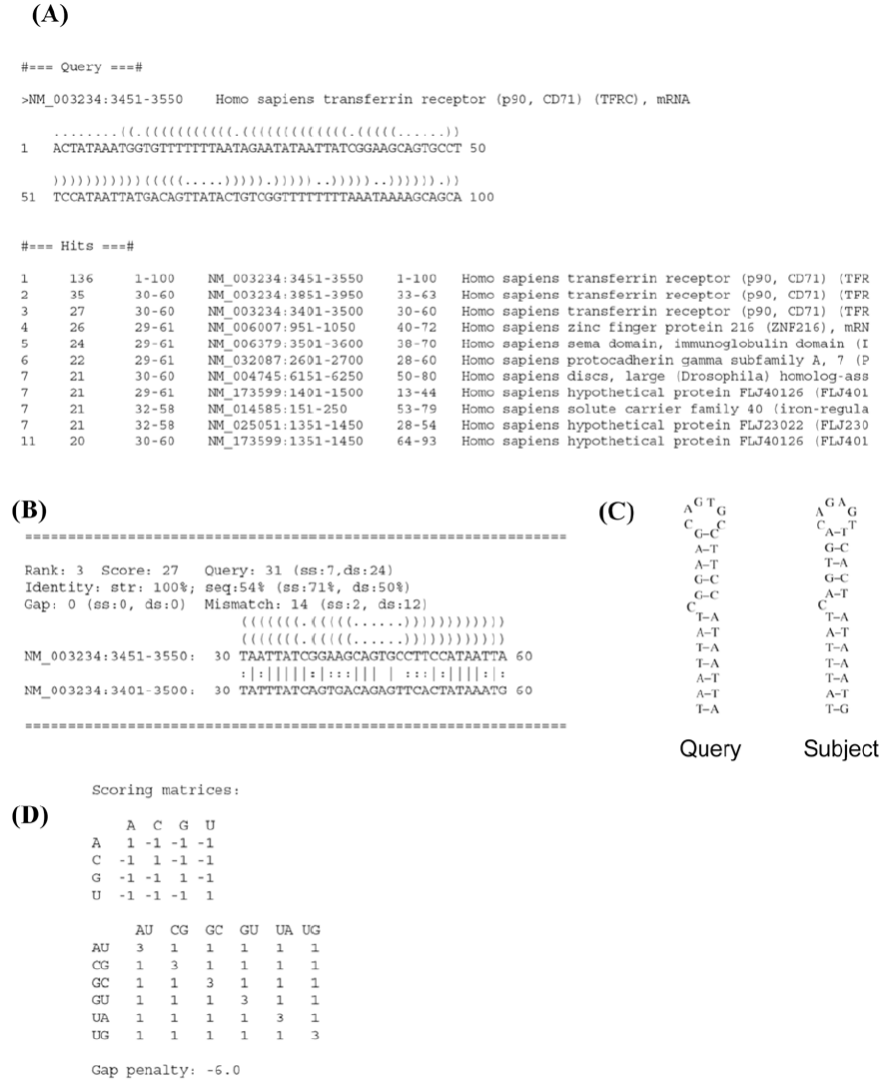
**Database search with an RNA structure containing an IRE motif. **A structure element (from base 3,451 to base 3,550) in the 3'UTR of human transferrin receptor (NM_003234) was used as a query to search the UTR structure database. (A) The output from RSmatch showing the top 11 hits. The six columns in the ''Hits'' section are, from left to right, rank, alignment score, region in the query, name of the hit, region in the hit, and annotation of the hit respectively. (B) A pairwise alignment of the query structure and a hit structure (NM_003234:3401-3500), which is the region from base 3,401 to base 3,500 of transferrin receptor (NM_003234). The sequence length is shown after "Query" on the first line: a 31 nt long query sequence containing 7 nt in ss region and 24 nt in ds region. Numbers after "Identity" on the second line are percentages of identity of secondary structure (100%), and primary sequence (54%). The latter is further decomposed into two numbers indicating the sequence identity in ss region (71%) and ds region (50%) respectively. The number of gaps in the overall alignment is shown after "Gap", followed by the number of gaps in ss region and ds region, both shown in parenthesis. The same format is used for nucleotide mismatches. Alignments of both structure and sequence are given, where "|" indicates identical nucleotides in either ss region or ds region, and ":" indicates identical secondary structures with different sequences. RNA structures are presented as follows: nested parentheses are used for base pairs and dots are used for nucleotides in ss regions. (C) The RNA structures corresponding to the query and the subject (hit) structure in (B). (D) Scoring matrices and the gap penalty used in the search. T and U are used interchangeably in this study.

We tested our program with a query sequence containing an iron response element (IRE). The IRE motif is a bipartite stem-loop structure containing ~30 nucleotides. Two alternative types of IREs have been found, which differ in the middle region [3]. Type I has a bulge, whereas type II has a small internal loop. IREs have been found in both 5' and 3' UTRs of genes that are involved in iron homeostasis in higher eukaryotic species. They interact with iron regulatory proteins (IRPs) and play a role in RNA stability and translation. Using a subsequence in the 3'UTR of transferrin receptor (NM_003234) that contains an IRE motif, we searched the UTR structure database described in Implementation. A list of top hits is shown in Figure 3. The best hit of the search is the query structure itself, as expected. Other regions of the same mRNA and regions of other RNAs are also found to have homologous structures with the query. As clearly shown in the result, the region containing the IRE motif, which is from about the 30th nucleotide to about the 60th nucleotide of the query structure, has been located by the RSmatch program, indicating that a local optimal alignment has been achieved. Among the top 10 hits, several sequences are known to have IREs, such as several regions in the 3'UTR of transferrin receptor (NM_003234) and the 5'UTR of solute carrier family 40 protein (NM_014585). Other top hits have not been shown so far to have IREs. It is not known if some of them are novel IRE-containing RNAs and the definitive answer will await wet lab validation. The output shows detailed alignment and related information, including the numbers of bases in the single-stranded and double-stranded regions, and the percentages of identity in single-stranded and double-stranded regions.

**Figure 4:**
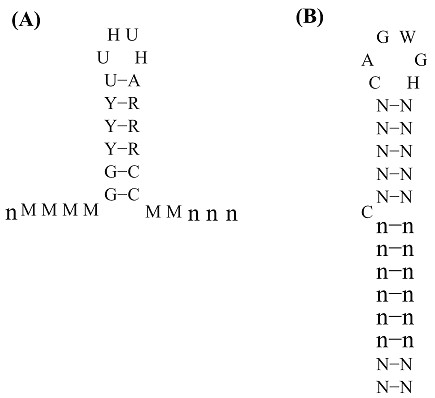
**The two pattern-based RNA structures used in this study. **(A) Histone 3'-UTR (HSL3) motif. (B) Iron Response Element (IRE) motif. A wildcard, represented by a lowercase letter n, is allowed to appear in a motif. When matching the motif with an RNA secondary structure, the wildcard in the motif can be instantiated into zero or one nucleotide in the secondary structure at no cost. Wildcards are used in places where the length of a region, either single-stranded or double-stranded, is variable. For example, the 5' flanking tail of HSL3 can be 4 or 5 nt long, and the lower part of the stem region of IRE can be 2 to 8 nt long.

RSmatch can also accept pattern-based RNA structures (sometimes called descriptors) to search a structure database. Since a pattern-based search method has an intrinsic primitive scoring scheme by using degenerate bases, we used simplified binary matrices as the equivalent to score an alignment. In the matrices, the match of a pair of structure components (single bases or base pairs), including those containing degenerated bases, was given a score of 1, a mismatch was penalized by a score of -1, and the atomic gap penalty *u *was set to -3. To allow variability in single-stranded and/or double-stranded regions for a structure pattern, we introduced a wildcard "n (lower case)" to represent optional single base component ("n") and base pair component ("n-n"). The meaning of "n" is identical to the IUB code "N" except that the matching score for both structure components "n" and "n-n" is always zero regardless of whether they are aligned with a structure component or a gap. Two RNA motifs were used to test our method, namely a histone 3'UTR stem-loop structure (HSL3) and IRE. HSL3, which resides in the 3'UTR region of histone mRNAs, has a typical stem loop structure with two flanking tails (Figure 4A). Both the stem and the flanking sequences are important to bind with a stem-loop binding protein (SLBP), which controls the pre-mRNA processing and stability of histone mRNAs [42]. In contrast to the HSL3 motif, IRE is relatively flexible in length and in nucleotide composition in its stem region (Figure 4B). We compared our program with PatSearch [35], a widely used tool that searches a sequence database for sequence and structure patterns.

Using the HSL3 motif and UTR sequence database, PatSearch found 55 hits whose locations were presented in Table 2. Among them, one is a false positive (NM_014372, ring finger protein 11, Table 2). Therefore the specificity (98.2%) of PatSearch is very high. This is attributable to the precise specification of the HSL3 pattern. However, if a pattern description is too precise, it may lead to the "overfitting" problem. This problem prevents the tool from finding slightly divergent structures, thus lowering the tool's sensitivity. Indeed, several histone genes were not detected by PatSearch, including two histone genes (histone H4c NM_003542 and histone H4 NM_003548) which were found by RSmatch among its top 33 hits. Several other histone genes appeared among the top 184 hits of RSmatch (Table 2). This indicates that by gaining specificity, PatSearch loses sensitivity for HSL3. Since RSmatch gives a score to each alignment, different cutoffs can be used for selecting top hits (Table 3). It seems that newly detected true positives are heavily outnumbered by false positives as RSmatch relaxes its cutoff value. However, with some properly chosen cutoff, i.e. 12, RSmatch could still achieve a comparable specificity with PatSearch. One possible explanation of getting high false positives for RSmatch could be that, with respect to the particular case of the HSL3 motif, its secondary structure conformation might be too pervasive in RNA sequences to be used as a discriminative feature. This could point out a problem concerning RSmatch's current scoring matrices, which need to be fine tuned to improve the tool's specificity. Good tuning could be achieved by setting up the scoring matrices through learning from a training data set. One interesting observation, however, was that RSmatch and PatSearch agreed perfectly upon the HSL3 locations for almost all of the true positives they found.

Using the IRE motif, we performed further comparisons between RSmatch and three other tools: PatSearch [43], stemloc [32] and Rsearch [31]. We used default parameters for Rsearch; for stemloc, the fold envelope was set to 1000. Instead of using the large UTR structure database described in Implementation we constructed a small test data set to expedite the comparison process. First, we used PatSearch to search human UTR sequences for IRE motifs. Then for each hit sequence we selected its corresponding mRNA's 3' or 5' UTR sequence. Following the same folding process as discussed in Implementation, we folded these UTR sequences to form the test data set. Totally, PatSearch found 27 hits, among which 9 were known true positives. Therefore PatSearch's specificity was ~33%. These hits were from 23 distinct mRNA sequences. We assumed that PatSearch had a 100% sensitivity. We extracted the 5' and 3' UTR sequences from the 23 distinct mRNAs and obtained 46 UTR sequences. We then folded the 46 UTR sequences to get a small test data set, which contained 1196 structures. Using a known IRE-containing structure (NM_000032), which was one of the 9 true positives found by PatSearch, as the query, we searched the small test data set. Table 4 shows the results we obtained. Since Rsearch accepts sequences only, it was tested using only the primary sequence information in the test data set.

Except for the IRE-containing structure NM_001098, which was one of the 9 true positives found by PatSearch, and the query itself (NM_000032), all tools agreed on the IRE locations for the other seven true positives without salient discrepancy. It was found that NM_001098 was not properly folded to exhibit the existence of the IRE motif. RSmatch has the best specificity by ranking all seven true positives within its top 8 hits with only one false positive (NM_032484). Rsearch is close to RSmatch by ranking all seven true positives within its top 8 hits with one false positive (NM_003672). In contrast, stemloc gives five false positives within its top 10 hits. Setting different cutoff values yields different specificity and sensitivity for each tool. The point of balanced specificity and sensitivity appears at the cutoff value of 8 for all three tools. With this cutoff value, the specificity of RSmatch and Rsearch tied at 7/8 × 100% = 87.5%. This is better than the specificity of PatSearch (33%) and the specificity of stemloc (~50%). The sensitivity of RSmatch, Rsearch and stemloc is 87.5%, 87.5% and 50% respectively. It is worth noting that RSmatch runs ~30% faster than Rsearch; it took Rsearch 34 seconds to search the whole data set of 1196 structures while RSmatch used only 23 seconds. Consequently, RSmatch would be suitable for analyzing large data sets. It should also be pointed out that RSmatch permits wildcards in database searching and structure matching, which are not supported by Rsearch or stemloc.

### Performance on complex structures

We further tested how accurate RSmatch is for complex structures. To this end, we downloaded RNA structures and sequences from the Rfam database (see Implementation). We used 64 RNA structure families, each of which has more than 10 seed sequences and has the consensus sequence length less than 250 nucleotides. For each RNA structure family, we randomly selected a structure and searched against 10 randomly selected sequences belonging to the same family. To reflect real world scenarios, we extended RNA sequences at both 5' and 3' ends so that the length of a subject sequence is three times that of the original one. To ensure that the folded structures are long enough to fully contain the structure being investigated, we required the moving window size to be 1.5 times the length of the query RNA sequence. Furthermore, to include suboptimal structures, we used all structures with free energy within 1.5 kcal/mol above the minimum one. Compared with HSL3 and IRE, the 64 query structures we used were much more complex, with average length of ~120 nt and more than 70% of them comprised of nested loops and conjunctions.

**Figure 5:**
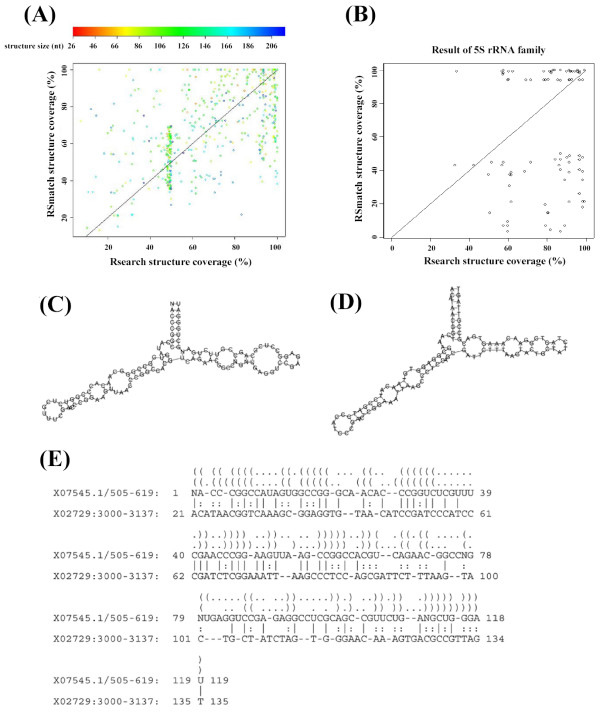
**Performance comparison of Rsearch and RSmatch and an alignment of two 5S rRNAs. **(A) Performance comparison for 64 RNA families. Different colors are applied to represent structures of different sizes. Each point corresponds to one alignment between a query structure and a subject structure. The x-axis is the percent of coverage by Rsearch and y-axis is the percent of coverage by RSmatch. (B) Performance comparison of 5S rRNA. A 5S rRNA was randomly chosen as the query structure and ten others as the subject sequences. The median value of the ten structure coverage values was then calculated. This process was repeated 100 times to generate 100 points for the graph. Therefore, each point represents one particular query structure. An example alignment of two 5S rRNA was shown: (C) the query structure is X07545/505-619; (D) the subject RNA is X02729; and (E) the detailed alignment by RSmatch.

To assess the accuracy, we used a measure called structure coverage, denoted as *p*, which is calculated by the following formula: *p *= |Q_align_|/max(|Q|, |S_align_|), where |Q_align_| and |S_align_| are the lengths of aligned portion of query RNA and subject RNA, respectively, and |Q| is the length of query RNA sequence. As shown in Figure 5A, even though Rsearch has slightly more points clustered around high coverage (90–100%), the overall difference between RSmatch and Rsearch is not significant. In addition, the difference between RSmatch and Rsearch do not seem to be related to structure size or complexity. This result indicates that RSmatch has the ability to process complex structures.

We also selected 5S rRNA for further detailed tests. 5S rRNA has a length of ~120 nt, which contains several types of RNA structures, including hairpin, internal loop, bulge, and junction. There are 602 sequences in the 5S rRNA family, allowing us to carry out a thorough analysis. We randomly chose one 5S rRNA as query structure and ten others as subject sequences for alignment. This process was repeated 100 times. The performance comparison of Rsearch and RSmatch is shown in Figure 5B. For 5S rRNA, RSmatch outperforms Rsearch in discovering the complete structure more frequently. An exemplary alignment is shown in Figure 5C–5E.

### Running efficiency

**Figure 6:**
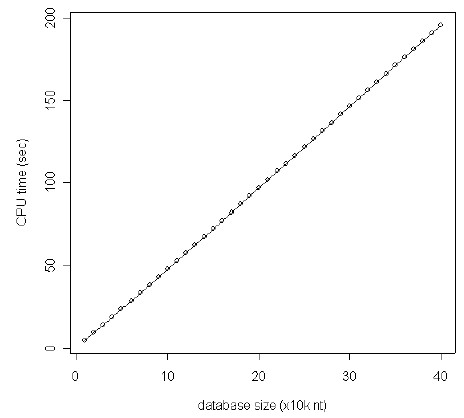
**CPU time versus database size. **From the 5S rRNA family, a randomly picked 5S rRNA was used as the query to search a structure database obtained by folding the rest seed sequences in the family. The program was run 10 times, and the average running time of each time is shown as a circle in the graph.

By dynamic programming, the running time of computing an alignment equals the number of writing operations needed to fill the scoring table. Thus the time complexity of RSmatch is *O(mn)*, where *m *(*n*, respectively) is the number of structure components in the query (subject, respectively) RNA structure. To test the scalability, we downloaded the seed sequences for 5S rRNA family from Rfam and randomly selected one annotated structure as the query while folding the rest sequences to prepare the structure database as discussed above (Figure 6). We plotted the RSmatch running time versus the database size. The program was run 10 times and the result is shown in Figure 6. The nearly perfect linear growth of the running time gives an empirical proof that the algorithm's time complexity is bounded by *O*(*nm*).

### Multiple structure alignment and iterative database search

**Figure 7:**
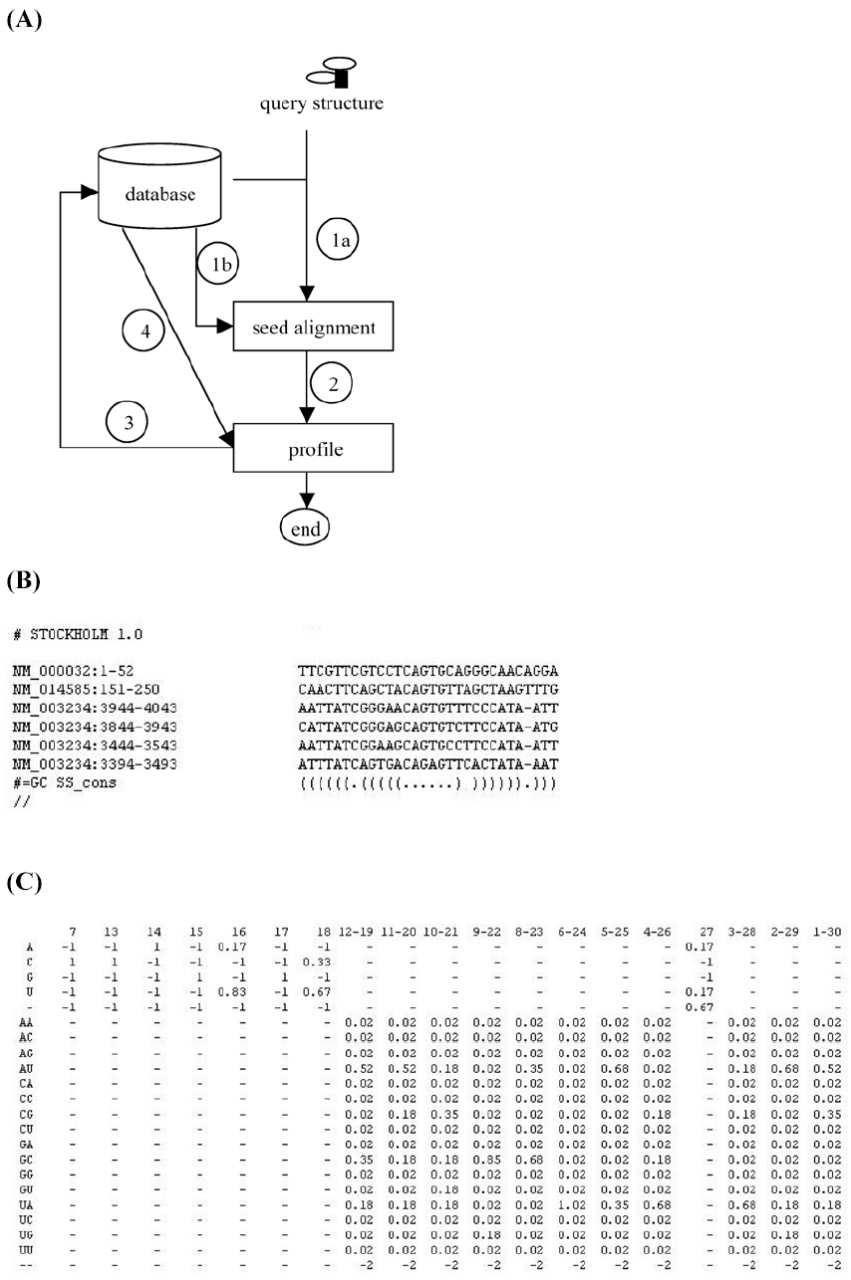
**Multiple structure alignment and iterative database search. **(A) Flowchart of multiple structure alignment and iterative database search. Step (1a) accepts a query structure to start an iterative database search; step (1b) processes a small database for multiple structure alignment; step (2) derives a profile from the seed alignment; step (3) uses the profile to conduct search; and step (4) updates the profile with new alignment. (B) Multiple structure alignment of several IRE structures. (C) PSSM of the multiple alignment of IRE in (B). Each column in the PSSM corresponds to the position of a structure component, either single base or base pair. Position of a single base is represented by the nucleotide number and position of a base pair is represented by two nucleotide numbers connected by a dash. For each column, the scores of individual structure components in that position are listed in rows where "-" means not applicable.

We also extended RSmatch algorithm to conduct multiple structure alignment. An example using IRE is shown in Figure 7. While the alignment algorithm is the same, the multiple alignment function uses a position-specific scoring matrix (PSSM, Figure 7C). For a given set of structures, the multiple alignment function first identifies the best alignment of two structures, and builds a PSSM. The PSSM is then used to search for the closest structure in the rest of the set. A flowchart of multiple structure alignment is shown in Figure 7A. If the alignment score of a structure to the PSSM is above a cutoff (user-defined), it is selected and its structure is used to update the PSSM. This step is iteratively conducted until no structures have alignment score above the cutoff. In a sense, this method employs an implicit guided hierarchical tree using the average value for joining nodes. As an example, from our human UTR database we selected 6 IRE-containing structures and randomly chose other 6 none-IRE structures to form a small dataset and run RSmatch against it. The output is shown in Figure 7B. The final result is in Stockholm format for multiple structure alignment. Conceivably, when the given set of structures is a large database, the multiple structure alignment function of RSmatch in effect conducts iterative search for finding similar structures.

## Discussion and conclusions

The work presented here is intended to provide an efficient tool to directly perform structure alignment and search of RNA secondary structure databases. Its capability to carry out multiple structure alignment and iterative database search can potentially be used to uncover RNA motifs *ab initio*. For example, one can use an RNA structure of interest to search an RNA structure database, and build PSSM iteratively to build an RNA motif, as demonstrated for IRE in this study (Figure 7).

RSmatch bears similarities to rna_align and RNAforester in that the structural particularities are either explicitly captured using hierarchical tree/forest structures or implicitly represented using arc-annotated structures. However, RSmatch differs from rna_align and RNAforester in two major aspects. First, RSmatch keeps structural consistence by only allowing single bases matched with single bases and base pairs matched with base pairs whereas rna_align and RNAforest do not impose this restriction. Second, RSmatch keeps the integrity of single-stranded regions by matching one with another, instead of breaking a single-stranded region into pieces and aligning them with different single-stranded regions. In addition, RSmatch has less time and space complexities than the other two tools.

The concept of circles introduced in this paper is reminiscent of the "k-loop" described in the classic RNA structure prediction paper [44]. The difference is that the circles can reflect the inter-base-pair relationship by focusing on two base-pairs at a time while the "k-loop" cannot. By organizing all circles into a hierarchy tree, we can capture the overall structural particularity. It should also be pointed that there is a major difference between the hierarchy tree introduced here and the parse trees of SCFG [28]. The hierarchy tree is constructed from circles and aims to obtain the panorama of the secondary structure of RNA at a higher level than that of the SCFG parse tree, while detailed information is still available within each circle in the tree. With the introduction of partial structures, this two-level structure modeling (intra- and inter- circles) allows us to develop an efficient algorithm that runs at time *O(mn) *as we have shown in the paper.

Our program takes full advantage of structure prediction techniques. It separates RNA folding from structure alignment. Simultaneous RNA folding and alignment is believed to be the optimal solution for both finding the right structure and locating homologous sub-structures of RNAs [17]. Unfortunately, it is computationally prohibitive for even a moderate number of RNAs. Some improvements have been proposed, but extensive computing time still makes them infeasible for database searches [18,19]. By separating the process into two steps, we greatly enhanced the computing efficiency, making it possible to process a large-scale pre-folded RNA structure database for homologous motifs. However, a drawback of using pre-folded RNAs is that the prediction tools may not produce correct RNA structures, as observed in our experiments. It is estimated that the RNA folding programs solely based on thermodynamic properties of RNA can correctly predict RNA structures with about 70% of chance [45]. Secondly, higher complex structures, such as pseudoknots, cannot be predicted in most commonly used programs, including the Vienna RNA package used in this study. A solution to removing the first drawback is to choose suboptimal structures in addition to the optimal one to increase the chance of obtaining correct structures. It has been reported that using suboptimal structures whose thermodynamic free energies are within 2% of that of the optimal one can greatly improve the structure prediction of RNA [44]. In our IRE experiments, we found that the predicted structure for NM_001098/1–23 did not exhibit the existence of an IRE motif. By relaxing the free energy range, we finally detected the IRE motif from one suboptimal structure whose free energy was 1.7 kcal/mol higher than the optimal one. Because of the computing efficiency of our program, an increase of the number of RNA structures does not impose big burden on database searching (data not shown). The cost will be at the database building stage, which is however done only once.

The moving window approach we used to extract and fold subsequences was aimed to make the folding process more accurate and efficient. This is because RNA folding programs are known to have pronounced difficulties in correctly predicting large RNA structures. Furthermore, predicting the structure for a long sequence takes much longer time than predicting structures for its subsequences. Another advantage of using the moving window method is that small motifs falling in the overlapped subsequences could be folded twice, increasing the chance of their being detected.

Pattern-based tools, such as PatSearch and RNAmotif, use descriptions of an RNA structure as queries to search a sequence database for similar structures. This type of search does not take into consideration the context of a hit sequence, which could influence the (sub)structure of the sequence. For example, as shown in our experimental results, PatSearch can achieve a satisfactorily high specificity when the structure of a pattern is not flexible and its description is relatively precise, such as the HSL3 motif. However, the sensitivity of PatSearch is low with rigid pattern descriptions. For relatively flexible structures, such as IREs, the specificity of PatSearch drops because it does not take into account the context in which a motif is located. On the other hand, using folded RNA structures, the proposed RSmatch tool overcomes these shortcomings with a high specificity, thus complementing the pattern-based tool. However, as also shown in our experimental results, the error existing in folding an RNA sequence (NM_001098) can lower the sensitivity of RSmatch. We suspect that the inaccuracy introduced by RNA folding could be a bottleneck for our technique in achieving a very high sensitivity.

Our scoring matrices for single-stranded and double-stranded regions and the gap penalty assignment are very primitive in the sense that they are not based on any probabilistic model or learned from any training data set. One interesting observation in our HSL3 experiment was that RSmatch did find most HSL3 sites correctly. However, the scoring scheme seemed not acute enough to filter out many false positives. Part of the problem is that there are not enough motifs that can be used to construct optimal scoring matrices. In fact, we also tested the matrices (RIBOSUM) proposed by Klein and Eddy, which were built upon small subunit ribosomal RNAs. We did not find any discernible difference in our HSL3 experiment, in which both matrices were used (data not shown). Another related question is whether different types of RNA, such as tRNA, rRNA, and UTRs, need their own scoring matrices. It is conceivable that large highly structured RNAs, such as rRNA, may be able to tolerate more mutations than short RNA motifs that occur in UTR regions. If so, using different scoring matrices for different types of RNAs will be warranted. Furthermore, it is possible that the mutation rate is different for nucleotides in different regions of an RNA motif. Therefore, PSSM might be more suitable in these cases. To this end, the iterative search function of RSmatch, which searches a database using PSSM, can be applied.

Motivated by the statistical methods of assessing results in sequence alignment [46], we tried to develop scores of our database search with known probabilistic distributions. The score distribution seemed close to be normal (data not shown). However since our scoring scheme is still at its preliminary stage and much is to be learned about the RNA structure database presented in the paper, we only presented search results in terms of ranking. More elaborate statistical assessment of the search results will be developed in the future.

## Availability and requirements

The RSmatch package has been implemented in Java and Perl and is freely available for academic use at  or .

## Authors' contributions

JL, JTW, and BT participate in the design of the algorithm. JL developed the software. JL and BT did the study with various RNA structures. JH tested the software and participated in the study of HSL3. JL, JTW and BT wrote the manuscript.

## Figures and Tables

**Table 1:** Performance comparison of RNA secondary structure tools

Tool Name	Running Time	Space Requirement	Reference
Sankoff^a^	*O*(N^6^)	*O*(N^4^)	[17]
FOLDALIGN^b^	*O*(N^4^)	*O*(N^4^)	[18]
RAGA^c^	*O*(M^2^N^3^)	*O*(M^2^N^2^)	[22]
rna_align^d^	min{*O*(MN^3^), *O*(M^3^N)}	*O*(MN^2^)	[26]
Dynalign^e^	*O*(M^3^N^3^)	*O*(M^2^N^2^)	[19]
stemloc^f^	*O*(LM)	N/A	[32]
Rsearch^g^	*O*(M^3^N)	*O*(M^3^)	[31]
RNAforester^h^	*O*(|F_1_||F_2_|deg(F_1_)deg(F_2_)(deg(F_1_) + deg(F_2_)))	*O*(|F_1_||F_2_|deg(F_1_)deg(F_2_))	[27]
CARNAC^i^	*O*(N^6^), *O*(N^2^)	*O*(N^4^), *O*(N^2^)	[20]
comRNA^j^	*O*(M^N^)	N/A	[21]

^a ^N is the average sequence length;

^b ^N is the average length of a given set of RNAs;

^c ^M and N are the lengths of the two given sequences;

^d ^M and N are the two sequence lengths;

^e ^M is the maximum distance allowed to match two nucleotides and N is the length of the shorter sequence;

^f ^L and M are the two RNA sequence lengths; only valid in extreme cases;

^g ^M is the query length and N is the subject sequence length;

^h ^|F_i_| is the number of nodes in forest F_i _and deg(F_i_) is the degree of F_i_;

^i ^N is the sequence length, theoretical time complexity of *O*(N^6^) could be significantly reduced to around *O*(N^2^) by pre-processing of the sequences, as noted by the authors [20].

^j ^M is the maximum number of stems examined and N is the number of total sequences under analysis. The comRNA's average run-time can be significantly improved by carefully chosen parameters, as noted by the authors [21].

**Table 2:** HSL3 motifs found by RSmatch and PatSearch^a,b^

RefSeq ID	Location by PatSearch^c^	Score of RSmatch	Location by RSmatch	Annotation
NM_002105	551–572	16	549–574	Hs H2A histone family, (H2AFX)
NM_003493	454–475	16	452–478	Hs histone 3, H3 (HIST3H3)
NM_003495	342–363	16	341–366	Hs histone 1, H4i (HIST1H4I)
NM_003509	445–466	16	444–469	Hs histone 1, H2ai (HIST1H2AI)
NM_003512	521–542	16	520–542	Hs histone 1, H2ac (HIST1H2AC)
NM_003517	413–434	16	412–437	Hs histone 2, H2ac (HIST2H2AC)
NM_003518	408–429	16	407–432	Hs histone 1, H2bg (HIST1H2BG)
NM_003519	429–450	16	428–450	Hs histone 1, H2bl (HIST1H2BL)
NM_003520	425–446	16	424–449	Hs histone 1, H2bn (HIST1H2BN)
NM_003522	406–427	16	405–427	Hs histone 1, H2bf (HIST1H2BF)
NM_003525	413–434	16	413–434	Hs histone 1, H2bi (HIST1H2BI)
NM_003526	414–435	16	413–435	Hs histone 1, H2bc (HIST1H2BC)
NM_003527	442–463	16	441–466	Hs histone 1, H2bo (HIST1H2BO)
NM_003528	476–497	16	475–500	Hs histone 2, H2be (HIST2H2BE)
NM_003530	443–464	16	442–467	Hs histone 1, H3d (HIST1H3D)
NM_003535	454–475	16	453–478	Hs histone 1, H3j (HIST1H3J)
NM_003537	445–466	16	444–469	Hs histone 1, H3b (HIST1H3B)
NM_003539	343–364	16	342–367	Hs histone 1, H4d (HIST1H4D)
NM_003546	340–361	16	339–364	Hs histone 1, H4l (HIST1H4L)
NM_005320	753–774	16	752–777	Hs histone 1, H1d (HIST1H1D)
NM_005325	733–754	16	732–757	Hs histone 1, H1a (HIST1H1A)
NM_021052	494–515	16	493–516	Hs histone 1, H2ae (HIST1H2AE)
NM_021059	483–504	16	483–504	Hs histone 2, H3c (HIST2H3C)
NM_021062	407–428	16	406–431	Hs histone 1, H2bb (HIST1H2BB)
NM_021063	463–484	16	462–484	Hs histone 1, H2bd (HIST1H2BD)
NM_021064	470–491	16	469–494	Hs histone 1, H2ag (HIST1H2AG)
NM_021066	414–435	16	413–435	Hs histone 1, H2aj (HIST1H2AJ)
NM_021968	331–352	16	330–355	Hs histone 1, H4j (HIST1H4J)
NM_170610	413–434	16	412–437	Hs histone 1, H2ba (HIST1H2BA)
NM_175055	428–449	16	427–450	Hs histone 3, H2bb (HIST3H2BB)
NM_003542	N/A^c^	14	365–390	Hs histone 1, H4c (HIST1H4C)
NM_003548	N/A	14	371–396	Hs histone 2, H4 (HIST2H4)
NM_021058	457–478	14	455–481	Hs histone 1, H2bj (HIST1H2BJ)
NM_003510	436–457	12	435–456	Hs histone 1, H2ak (HIST1H2AK)
NM_003511	446–467	12	445–466	Hs histone 1, H2al (HIST1H2AL)
NM_003514	463–484	12	462–483	Hs histone 1, H2am (HIST1H2AM)
NM_003516	510–531	12	509–530	Hs histone 2, H2aa (HIST2H2AA)
NM_003523	411–432	12	412–435	Hs histone 1, H2be (HIST1H2BE)
NM_003529	439–460	12	440–462	Hs histone 1, H3a (HIST1H3A)
NM_003536	449–470	12	448–469	Hs histone 1, H3h (HIST1H3H)
NM_005319	709–730	12	708–729	Hs histone 1, H1c (HIST1H1C)
NM_005322	766–787	12	767–787	Hs histone 1, H1b (HIST1H1B)
NM_021018	444–465	12	445–466	Hs histone 1, H3f (HIST1H3F)
NM_175054	389–410	12	388–409	Hs histone 4, H4 (HIST4H4)
NM_175065	425–446	12	424–445	Hs histone 2, H2ab (HIST2H2AB)
NM_033445	472–493	10	471–492	Hs histone 3, H2a (HIST3H2A)
NM_003513	452–473	8	454–476	Hs histone 1, H2ab (HIST1H2AB)
NM_003521	N/A	8	421–441	Hs histone 1, H2bm (HIST1H2BM)
NM_003524	401–422	8	400–420	Hs histone 1, H2bh (HIST1H2BH)
NM_003533	453–474	8	452–472	Hs histone 1, H3i (HIST1H3I)
NM_003534	N/A	8	442–462	Hs histone 1, H3g (HIST1H3G)
NM_003540	N/A	8	348–368	Hs histone 1, H4f (HIST1H4F)
NM_003541	331–352	8	330–350	Hs histone 1, H4k (HIST1H4K)
NM_003543	N/A	8	349–369	Hs histone 1, H4h (HIST1H4H)
NM_003545	N/A	8	352–372	Hs histone 1, H4e (HIST1H4E)
NM_170745	441–462	8	440–460	Hs histone 1, H2aa (HIST1H2AA)
NM_003531	435–456	4	438–459	Hs histone 1, H3c (HIST1H3C)
NM_003532	438–459	4	441–459	Hs histone 1, H3e (HIST1H3E)
NM_005323	701–722	-4	705–721	Hs histone 1, H1t (HIST1H1T)
NM_021065	436–457	-10	314–335	Hs histone 1, H2ad (HIST1H2AD)
NM_005321	761–782	-41	85–116	Hs histone 1, H1e (HIST1H1E)
NM_014372	1345–1366	-42	1381–1389	Hs ring finger protein 11 (RNF11)

^a^Items listed here include those found by PatSearch and those found by RSmatch using cutoff value of 8 that are related to histone genes.

^b^RSmatch gets 33 hits at cutoff value of 14 and gets 184 hits at cutoff value of 8.

^c^mRNAs that are not detected to have the HSL3 motif by PatSearch are marked with "N/A".

**Table 3:** Performance of RSmatch in the HSL3 experiment^a^

Cutoff Score	Selected Hits^b^	True Positives	Specificity	Sensitivity^c^
14	33	33	100.0%	53.2%
12	47	45	95.7%	72.6%
10	69	46	66.7%	74.2%
8	184	56	30.4%	90.3%

^a^PatSearch has a specificity of 98.2% and sensitivity of 87.1%.

^b^Hits whose scores are greater than or equal to the cutoff value used in this study are selected.

^c^Assume there are 62 mRNA structures containing the HSL3 motif, which include all histone mRNAs found by RSmatch and PatSearch.

**Table 4:** IRE experiment results

True Positive	RefSeq ID	Location by PatSearch	RSmatch			Rsearch			stemloc		
			
			Location	Score	Rank	Location	Score	Rank	Location	Score	Rank
x	NM_000032^a^	13–35	-	-	-	-	-	-	-	-	-
x	NM_014585	203–229	202–231	21	1	202–231	34.11	1	202–226	13.021	5
x	NM_003234	3479–3511	3484–3506	19	2	3480–3510	31.42	2	3486–3503	15.936	2
x	NM_003234	3883–3913	3887–3909	17	3	3876–3925	27.80	6	3889–3906	10.914	7
x	NM_003234	3950–3976	3952–3974	17	3	3952–3974	25.50	10	3954–3971	10.476	9
x	NM_003234	3996–4024	3999–4021	17	3	3999–4022	28.53	4	4042–4048	1.149	25
x	NM_000146	19–41	20–40	16	6	7–51	27.84	5	17–41	8.574	12
	NM_032484	2353–2376	2358–2373	13	7	2355–2377	22.26	11	2354–2375	16.411	1
x	NM_003234	3429–3461	3434–3456	13	7	3433–3458	26.40	8	3436–3453	6.218	15
	NM_018992	2182–2205	2186–2202	12	9	2186–2202	18.43	19	2186–2202	11.459	6
	NM_003449	2160–2180	2163–2178	11	10	2160–2180	26.27	9	2161–2181	13.198	4
	NM_002081	3449–3469	3452–3467	11	10	3446–3472	20.47	17	3450–3470	8.290	14
	NM_173649	1371–1398	1431–1446	7	12	1372–1398	18.83	18	1376–1396	8.493	13
	NM_033337	1202–1226	1253–1257	5	13	1202–1227	21.52	14	1202–1227	4.540	18
	NM_001234	1106–1130	1157–1161	5	13	1106–1131	21.52	15	1106–11331	4.540	19
	NM_153706	174–194	108–119	5	13	171–198	17.49	20	219–234	4.400	20
	NM_003607	6892–6914	6851–6854	4	16	6892–6914	21.98	12	6930–6950	10.827	8
	NM_002086	82–102	126–129	4	16	94–117	16.48	22	101–125	2.833	22
	NM_012256	2594–2617	2571–2574	4	16	2536–2570	20.76	16	2590–2606	1.770	24
x	NM_001098	1–23	17–19	3	19	1–23	30.67	3	3–20	14.185	3
	NM_006731	4439–4465	4487–4489	3	19	4442–4462	21.65	13	4443–4460	9.920	10
	NM_003672	2556–2576	2592–2594	3	19	2547–2587	26.44	7	2558–2574	9.049	11
	NM_018234	2038–2058	2176–2178	3	19	2035–2061	14.11	24	2046–2058	4.986	16
	NM_024076	1799–1822	1816–1818	3	19	1800–1821	15.00	23	1832–1850	4.876	17
	NM_000877	3274–3294	3336–3338	3	19	3275–3293	13.81	25	3302–3321	3.182	21
	NM_003675	2–27	27–31	3	19	1–29	16.74	21	21–31	1.980	23
	NM_032323	1924–1944	1990–1992	3	19	1925–1943	12.43	26	1928–1948	0.678	26

^a^NM_000032 is used as the query structure for RSmatch, Rsearch, and stemloc. Thus there is no value (shown as "-").
